# Navigating Treble Clef Aorta: A Challenge for Transfemoral Transcatheter Aortic Valve Replacement—A Case Report

**DOI:** 10.1016/j.jscai.2024.102492

**Published:** 2024-12-31

**Authors:** Helen S. Anwar, José M. Montero-Cabezas, Frank Van Der Kley

**Affiliations:** a Department of Cardiology, Leiden University Medical Center, Leiden, the Netherlands; b Department of Cardiovascular Medicine, Assiut University Heart Hospital, Assiut University, Assiut, Egypt

**Keywords:** aortic stenosis, tortuous treble clef aorta, transfemoral transcatheter aortic valve replacement

## Abstract

Transcatheter aortic valve replacement (TAVR) has emerged as a widely approved intervention for managing severe aortic stenosis. As the number of patients undergoing TAVR continues to rise, certain cases present unique challenges, particularly in relation to vascular access. This report highlights a case of successful transfemoral TAVR performed in a patient with an exceptionally tortuous and elongated aortic arch, characterized by significant curvature reminiscent of the treble clef musical symbol.

Tortuous aorta poses unique challenges during transcatheter aortic valve replacement (TAVR) procedure. The complexities of navigating highly twisted and curved vessels can hinder device delivery, increase the risk of complications such as vascular injury or device malposition, and lead to longer procedural time.^1^^,^^2^ Severe tortuosity of the access route can affect guide wire manipulation and increase the risk of left ventricular perforation.^3^ Advanced imaging techniques and preprocedural planning are crucial to navigate the complex anatomy, and specialized tools and skills, or alternative access routes, may be considered to ensure procedural success and patient safety.

## Case presentation

An 84-year-old man with symptomatic severe aortic stenosis was referred to our institution for aortic valve intervention. Our patient had multiple cardiovascular risk factors (diabetes, hypertension, and dyslipidemia), previous percutaneous coronary intervention, and several comorbidities (a history of right mammary cancer and mastectomy, diverticulosis and left hemicolectomy, and right hip replacement). The patient’s surgical risk was high (EuroSCORE I logistic of 10.11%), and TAVR was discussed as the best and only interventional option for him.

**Figure 1:**
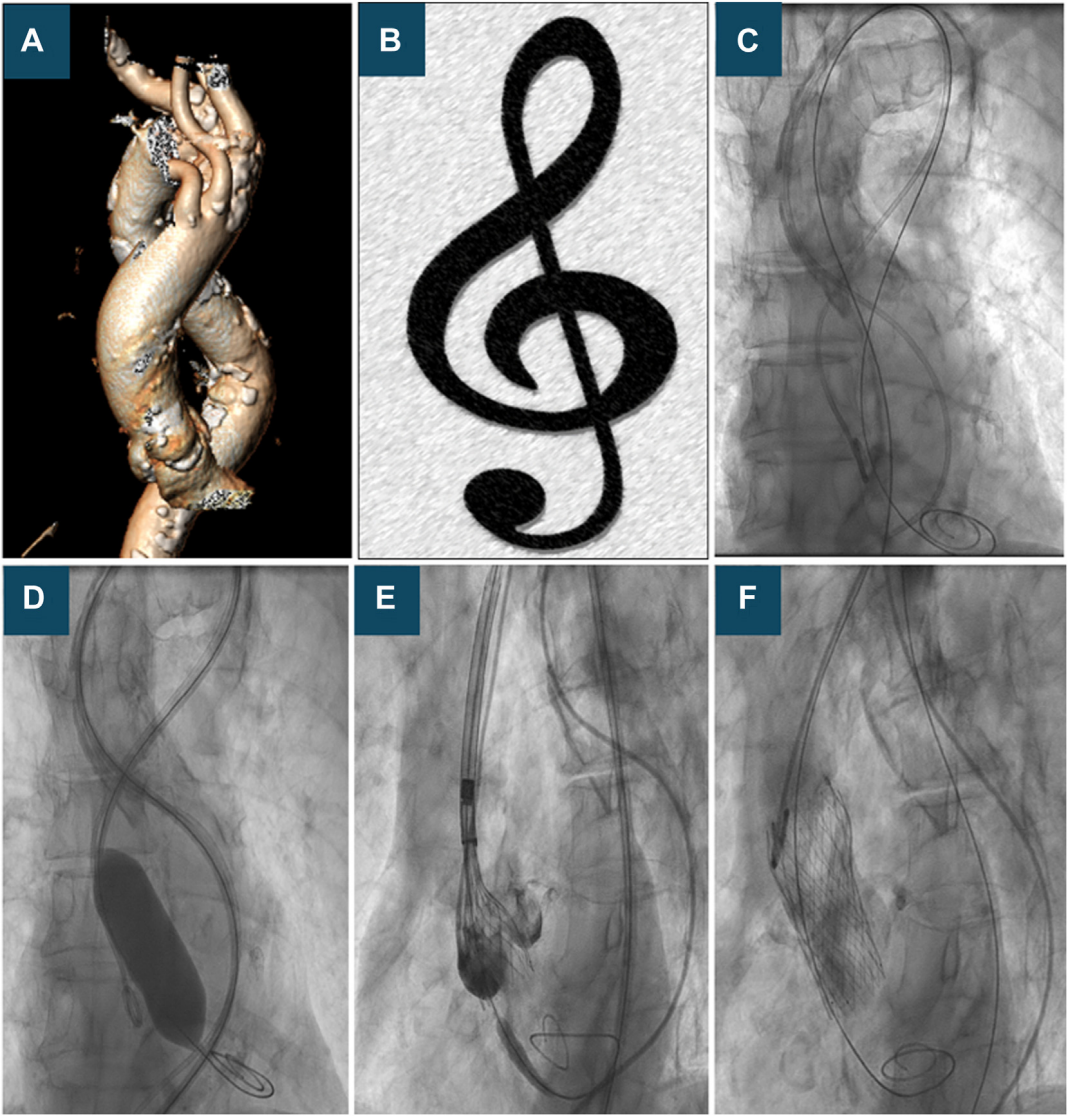
**Treble clef aorta: 3D multidetector computed tomography reconstruction and fluoroscopic imaging showed the extremely tortuous and elongated aortic arch resembling the Treble clef musical sign (A-C)**. Fluoroscopic imaging of transcatheter aortic valve replacement (D-F).

During the pre-TAVR multidetector computed tomography (MDCT) workup, we found extremely diseased, tortuous, and elongated descending thoracic aorta and aortic arch resembling a “treble clef” on the reconstructed images (Figure 1A, B). Transaxillary TAVR was less favorable due to a porcelain aorta and an aberrant subclavian artery (arteria lusoria). Our center had little experience with transcarotid TAVR, so transfemoral TAVR was accepted with this challenging anatomy. Cerebral protection device (Sentinel; Boston Scientific) was discussed but deemed not applicable due to arteria lusoria.

The TAVR procedure was done under local anesthesia with an ultrasound-guided puncture using a single right groin access. A14-F sheath (Cook) was placed in the descending aorta over the available super stiff INNOWI SX TAVR wire (SYMEDRIX GmbH) giving better support in this tortuous anatomy (Figure 1C). Valvuloplasty was done with a 22.0-mm VASC III balloon (OSYPKA AG) (Figure 1D). Medtronic Evolut FX valve was chosen owing to the great flexibility of the delivery system and for the superior benefit of recapture and retrieval of the valve. An Evolut FX 26.0-mm valve (Medtronic) was advanced into the aorta, which was very technically challenging, requiring extensive manipulation of the delivery system to negotiate the aortic arch, consisting of in back-and-forth movements and modification of the orientation of the wire position to facilitate the passage of the delivery system toward the aortic valve. Eventually, we succeeded, and the valve was correctly aligned and successfully deployed under accelerated pacing (Figure 1E, F; Supplemental Video 1).

Both final angiography and transthoracic echocardiography demonstrated good valve positioning and transvalvular gradient, with trace paravalvular leakage. At the end of the procedure, an aortogram was performed, which did not show any evidence of aortic injury. No other complications such as conduction defect or stroke were detected.

## Discussion

TAVR has become an established technique to treat patients with severe aortic stenosis aged older than 75 years.^4^ The recommendations are now extended to more patients even with moderate or low surgical risk after the favorable outcomes shown by multiple trials.^5^^,^^6^ Meticulous preprocedural planning is pivotal for the success of TAVR procedures. The primary objectives of this planning are to determine the most suitable vascular access route and conduct a comprehensive anatomical assessment of the aortic valve and aortic root. These evaluations are critical for selecting the appropriate valve type and size.^7^ Preprocedural multimodality imaging including echocardiography, angiography, and MDCT is essential in addressing these objectives, with MDCT playing a crucial role in precisely assessing the vascular access site.

Our patient’s pre-TAVR workup revealed an extremely tortuous and elongated aortic arch and descending thoracic aorta. The tortuous aorta has been described in previous literature,^8^ but an extremely tortuous and elongated aortic arch with arteria lusoria has not been stated before. This type of abnormality increases the challenges for both transfemoral and trans-axillary access.

## Conclusions

The precise and proper pre-TAVR MDCT-based planning makes performing transfemoral TAVR in even the most complex anatomical scenarios feasible using tailored techniques.
